# Endomucin prevents leukocyte–endothelial cell adhesion and has a critical role under resting and inflammatory conditions

**DOI:** 10.1038/ncomms10363

**Published:** 2016-02-02

**Authors:** Alisar Zahr, Pilar Alcaide, Jinling Yang, Alexander Jones, Meredith Gregory, Nathaniel G. dela Paz, Sunita Patel-Hett, Tania Nevers, Adarsha Koirala, Francis W. Luscinskas, Magali Saint-Geniez, Bruce Ksander, Patricia A. D'Amore, Pablo Argüeso

**Affiliations:** 1 Schepens Eye Research Institute/Massachusetts Eye and Ear and Department of Ophthalmology, Harvard Medical School, 20 Staniford Street, Boston, Massachusetts 02114, USA; 2 Brigham and Women's Hospital, Harvard Medical School, 77 Avenue Louis Pasteur, Boston, Massachusetts 02115, USA; 3 Department of Pathology, Harvard Medical School, 25 Shattuck Street, Boston, Massachusetts 02115, USA

## Abstract

Endomucin is a membrane-bound glycoprotein expressed luminally by endothelial cells that line postcapillary venules, a primary site of leukocyte recruitment during inflammation. Here we show that endomucin abrogation on quiescent endothelial cells enables neutrophils to adhere firmly, via LFA-1-mediated binding to ICAM-1 constitutively expressed by endothelial cells. Moreover, TNF-α stimulation downregulates cell surface expression of endomucin concurrent with increased expression of adhesion molecules. Adenovirus-mediated expression of endomucin under inflammatory conditions prevents neutrophil adhesion *in vitro* and reduces the infiltration of CD45^+^ and NIMP-R14^+^ cells *in vivo*. These results indicate that endomucin prevents leukocyte contact with adhesion molecules in non-inflamed tissues and that downregulation of endomucin is critical to facilitate adhesion of leukocytes into inflamed tissues.

## Abstract (web summary)

Endomucin is expressed by endothelial cells that line postcapillary venules—the site of leukocyte recruitment during inflammation. Zahr *et al.* show that endomucin is an anti-adhesive molecule that is downregulated by the cytokine TNF-a and thereby helps in the transition from a quiescent to a pro-adhesive inflamed endothelium.

## Graphical abstract

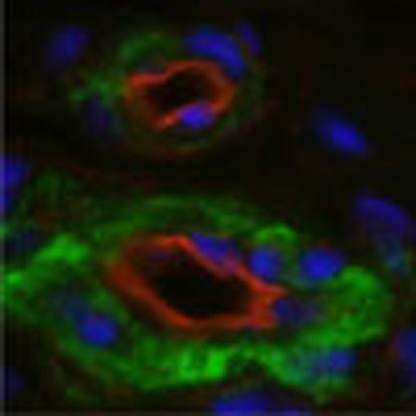

A primary function of the endothelium in the non-inflamed steady state is to maintain an anti-adhesive surface by regulating and inhibiting coagulation, and by resisting the adhesion of blood leukocytes^1^,^2^,^3^,^4^. It is well documented that activation of the endothelium in response to injury or infection leads to the upregulation of adhesion molecules and chemokines that localize the recruitment of blood leukocytes to specific tissues and organs^5^,^6^. Postcapillary venules, with their low laminar shear stress and sparse mural cell investment, are the primary site for the recruitment of leukocytes^7^,^8^. This response is comprised of a cascade of distinct sequential events including leukocyte tethering and rolling, firm adhesion and transmigration^6^.

The majority of the work conducted to understand the molecular regulation of leukocyte–endothelial interactions has focused on adhesion molecules and chemokines responsible for recruitment of leukocytes to the activated endothelium. These include E-selectin and P-selectin, which interact with P-selectin glycoprotein ligand 1 (PSGL1) and other glycosylated ligands on leukocytes; VCAM-1, which binds to very late antigen 4 (VLA4; also known as α_4_β_1_-integrin); and ICAM-1, which interacts with lymphocyte function-associated antigen 1 (LFA-1; also known as α_L_β_2_-integrin)^9^. Cytokines, bacterial endotoxins, hemodynamic factors and viruses predispose the surface endothelium to local thrombosis, loss of vessel barrier function and leukocyte recruitment. *In vitro* and *in vivo* studies have illustrated that leukocyte adhesion and crawling is initiated by activation of the endothelium by cytokines such as tumour necrosis factor alpha (TNF-α), interleukin-1 alpha and beta (IL-1α,β) and interferon gamma (IFN-γ)^10^. In contrast to adhesion molecules, much less attention has been focused on molecules that might prevent adhesive tethering of leukocytes or modulate their rolling without adhesion. This is particularly important since ICAM-1 is expressed on quiescent or ‘non'-activated endothelial cells^1^,^11^.

Glycoproteins in the endothelial glycocalyx have been shown to block adhesion of blood cells^12^. The endothelial glycocalyx is a negatively charged, organized meshwork composed of proteoglycans and their negatively charged glycosaminoglycans, glycoproteins bearing terminal sialic acids and associated plasma proteins^13^. Heparan sulfate proteoglycans, which comprise 50–90% of the surface glycosaminoglycans, are cleaved as part of the response to inflammation^14^, presumably to facilitate the association of leukocytes with the endothelial surface^15^,^16^. Sialomucins, such as CD34—a marker of vascular endothelial cells—or CD43 on leukocytes, have been shown to both support and prevent cell adhesion either by increasing or decreasing surface expression, or by post-translational modification, which allows controlled *O*-linked glycosylation^17^,^18^.

Endomucin (EMCN) is a type I integral membrane *O*-sialoglycoprotein expressed on the surface of endothelium in venules and capillaries, but not on arterial endothelium of adult tissues^19^,^20^. The deduced protein sequence of both human and mouse EMCN long splice variant has 261 amino acid residues with an extracellular domain rich in serine and threonine residues^21^,^22^. *O*-Glycans attached to these residues are important for maintaining the highly extended and rigid structure of the protein, as clustered *O*-glycans induce the peptide core to adopt a stiff and extended conformation that prevents folding into a globular structure^23^, which has an important role in regulating cell–cell and cell–matrix interactions. In the present study, we identify a function of EMCN in resisting the adhesion of blood leukocytes. Mechanistically, we demonstrate that expression of EMCN opposes the binding of neutrophils to constitutively expressed ICAM-1 on non-activated endothelial cells. Moreover, we find that inflammatory cytokines decrease EMCN expression, and that overexpression of EMCN under inflammatory conditions prevents neutrophil adhesion *in vitro* and reduces the infiltration of CD45^+^ and NIMP-R14^+^ cells *in vivo*. These data establish a critical role for EMCN in preventing leukocyte adhesion within non-inflamed tissues, and indicate that downregulation of EMCN during inflammation is necessary to facilitate migration of inflammatory cells into targeted tissues.

## Results

### Venous and capillary endothelial cells express EMCN

**Figure 1:**
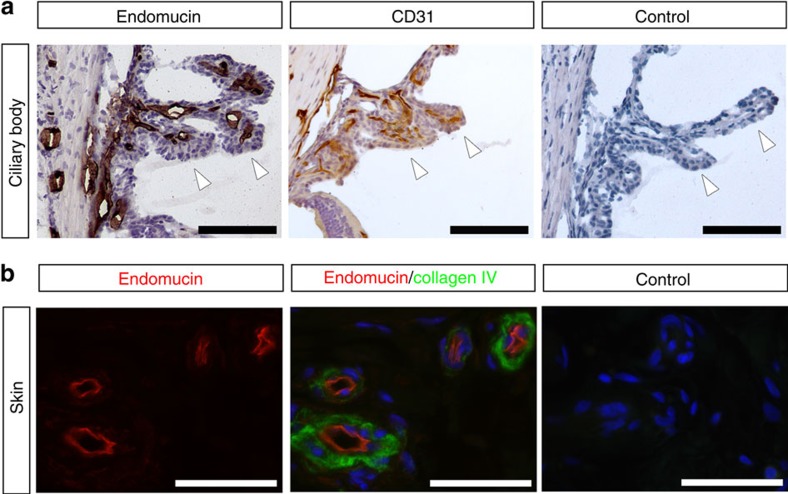
Venous and capillary endothelium expresses EMCN *in vivo*. (**a**) Tissues were dissected from adult C5BL6 mice, fixed in 4% paraformaldehyde, frozen in optimal cutting temperature (O.C.T.) compound and cut into 10 μm sections. EMCN localized to venules and capillaries of mouse ciliary bodies, and co-localized with CD31^+^ endothelial cells in the ciliary body. Ciliary processes in the posterior chamber are indicated with arrowheads. Scale bar, 100 μm. (**b**) In paraffin sections of normal human skin, EMCN (red) localized on the apical surface of venules marked by collagen IV (green). Nuclei were counterstained using DAPI (blue). Scale bar, 50 μm.

EMCN has been reported by us and others to localize to the luminal surface of venous, but not arterial, endothelium^19^,^20^. The ciliary body, which was examined in this study, is located in the anterior segment of the eye, and consists of ciliary muscles and ciliary processes, which contain fenestrated capillaries (Fig. 1a). In the mouse ciliary body, capillaries stained strongly for EMCN; notably, the staining co-localized with CD31^+^ endothelial cells. The expression of EMCN in human tissues correlated with the expression pattern in mice, and was observed on the apical surface of the endothelial cells of skin venules (Fig. 1b). Taken together, these results demonstrate the surface localization of EMCN in venous and capillary endothelium, which is consistent with previous reports^19^,^20^.

### EMCN depletion leads to increased neutrophil interactions

**Figure 2:**
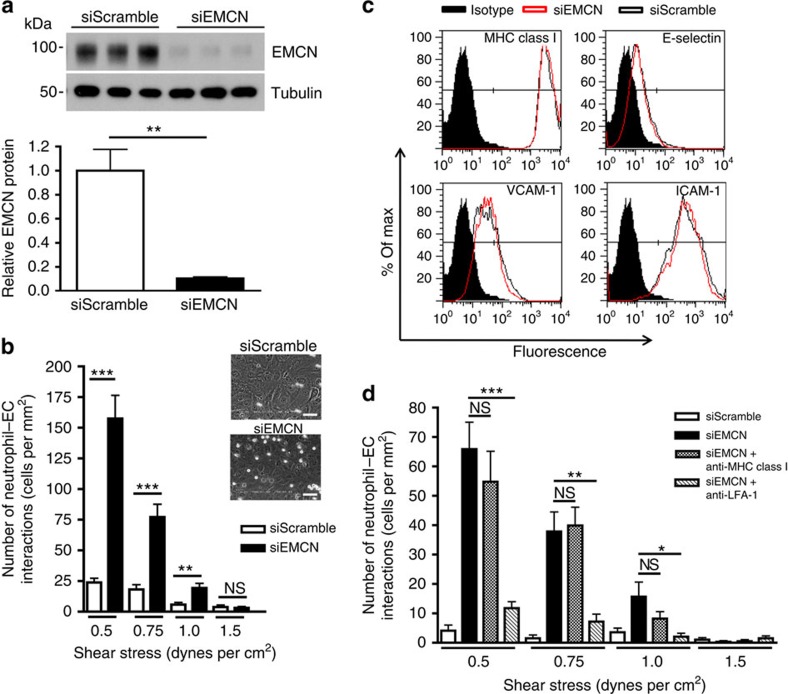
siRNA knockdown of EMCN leads to increased neutrophil–endothelial cell interactions. HUVECs seeded at 50% confluence were transfected with siRNA oligonucleotides targeted against EMCN. Analysis was performed 48 h after transfection on confluent endothelial cell monolayers. (**a**) siRNA led to a more than 80% knockdown of EMCN as determined by western blot in HUVECs. (**b**) Flow chamber studies revealed that at shear stresses of 0.5–1.0 dynes per cm^2^ there was >6-fold increase in adhesion of neutrophils compared to scramble-treated HUVECs. Representative images showing a freeze frame of neutrophils interacting with siScramble and siEMCN transfected HUVECs are shown. (**c**) Knockdown of EMCN did not alter the expression of pro-adhesive molecules, E-selectin, VCAM-1 or ICAM-1, as measured by flow cytometry. (**d**) Neutralizing antibody to LFA-1 reversed cell–cell interactions (including rolling and adherent cells) in siEMCN-treated HUVECs. Values for siRNA in (**a**) are expressed as mean±s.e.m., and results are representative of three independent experiments. Data in (**c**) represent one of the three independent experiments performed. In (**b**) and (**d**), results are representative of two to three human donors, and flow chamber experiments were performed in triplicate conditions, mean±s.e.m. Significance was determined for (**a**) using Student's *t*-test or (**b** and **d**) using one-way ANOVA followed by Newman–Keuls *post hoc* test. *P<0.05, **P<0.01 and ***P<0.001; NS, nonsignificant. Scale bar, 50 μm.

The apical localization of EMCN to venous endothelium suggests a possible role in cell–cell interactions. To examine the function of EMCN *in vitro*, a parallel-plate flow chamber was utilized to study the interactions between human neutrophils and human umbilical vein endothelial cells (HUVECs) under shear stresses that mimic those found in the postcapillary venous endothelium. Loss of function was achieved on HUVECs under static conditions using short interfering RNA (siRNA) to knockdown EMCN gene expression. siRNA against EMCN led to more than 80% reduction in EMCN protein (Fig. 2a). As shown in Fig. 2b, abrogation of EMCN led to a significant increase in the number of neutrophil–endothelial cell interactions at low shear stringency (0.5–1.0 dynes per cm^2^), that was not observed in the scramble siRNA control. These data demonstrate that under non-inflammatory quiescent conditions, EMCN blocks neutrophil interactions with the endothelium and in the absence of EMCN, neutrophils can bind resting endothelial cells.

Since non-inflamed HUVECs constitutively express basal levels of adhesion molecules, we examined whether knockdown of EMCN affected expression of E-selectin, VCAM-1 and ICAM-1. Flow cytometry analysis of these adhesion molecules revealed no change in their expression after EMCN knockdown (Fig. 2c). The higher level of ICAM-1 relative to E-selectin and VCAM-1 in non-activated endothelial cells is consistent with previous reports^1^,^11^. In light of these observations, we speculated that the siRNA knockdown of EMCN was enabling neutrophil interactions with the endothelium via the constitutively expressed ICAM-1. To test this possibility, neutrophils were pretreated with neutralizing monoclonal antibody (mAb) against LFA-1 (20 μg ml^−1^) or, as a negative control, anti-major histocompatibility complex (anti-MHC) class I mAb, and then allowed to interact under shear stress with HUVECs in which EMCN had been knocked down. Results revealed that blocking the interaction of neutrophils with ICAM-1 reduced neutrophil–endothelial cell interactions by more than 60% at low shear stress stringency (Fig. 2d). We conclude that the binding of neutrophils to constitutively expressed ICAM-1 on non-activated endothelial cells is opposed by robust EMCN expression.

### TNF-α suppresses EMCN expression

**Figure 3:**
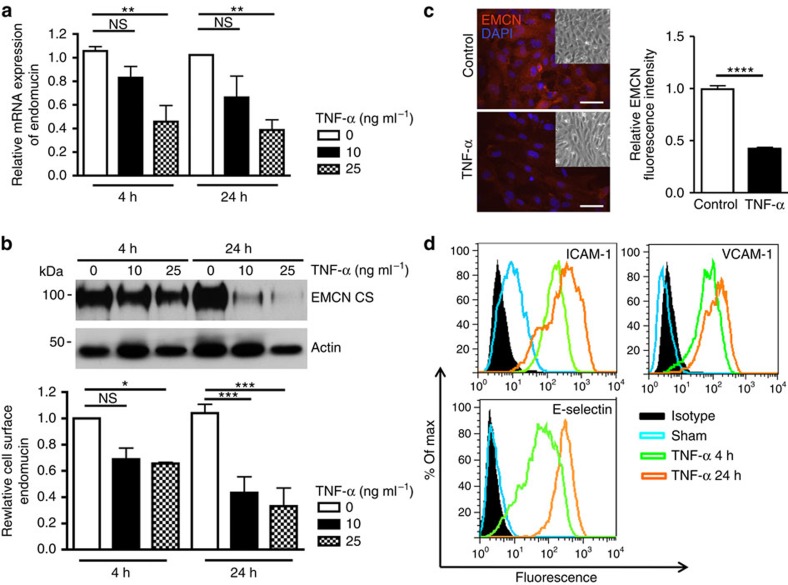
TNF-α inhibits EMCN expression *in vitro*. Confluent HUVECs were treated with TNF-α (10 and 25 ng ml^−1^) for 4 and 24 h after overnight serum starvation. mRNA, biotinylated protein and fixed cells were collected for analysis of (**a**) EMCN mRNA expression as determined by qRT–PCR (relative expression of EMCN as compared with GAPDH mRNA levels); (**b**) cell surface EMCN as determined by western blot; and (**c**) localization and quantification of EMCN (10 ng ml^−1^ for 24 h) fluorescence intensity as determined by immunofluorescence staining. Insets in (**c**) show corresponding phase-contrast images. The effect of TNF-α on the expression of pro-adhesive molecules, ICAM-1, VCAM-1 and E-selectin (**d**) were determined by flow cytometry. TNF-α treatment led to reduction of EMCN mRNA, cell surface EMCN and total EMCN while it upregulated pro-adhesive molecules ICAM-1, VCAM-1 and E-selectin. Values are expressed as mean±s.e.m. Data in (**d**) are plotted as percentage of max versus increasing fluorescence intensity. Results represent one of three independent experiments performed. Significance was determined for (**a** and **b**) using two-way ANOVA with Bonferroni's *post hoc* test or (**c**) using Student's *t*-test. *P<0.05, **P<0.01, ***P<0.001 and ****P<0.0001; CS, cell surface; NS, nonsignificant. Scale bar, 50 μm.

In light of the fact that leukocyte recruitment and infiltration occurs primarily in postcapillary venules during inflammation, we investigated the effect of cytokine treatment on endothelial expression of EMCN *in vitro*. Confluent HUVECs were stimulated with TNF-α (10 or 25 ng ml^−1^) for 4 and 24 h, and the expression of EMCN mRNA and cell surface protein were assessed by quantitative real-time PCR (qRT–PCR) and western blot analysis of biotinylated proteins, respectively. TNF-α treatment of HUVECs led to a dose-dependent decrease in EMCN mRNA at both 4 and 24 h (Fig. 3a). Similarly, TNF-α treatment of HUVECs led to a 35% reduction at 4 h, and a 70% reduction at 24 h, of cell surface EMCN in a dose-dependent manner (Fig. 3b). To account for variation in cell number after treatment with TNF-α, quantitative data on EMCN mRNA and cell surface protein levels were normalized to housekeeping controls GAPDH and actin, respectively. The TNF-α-induced reduction of EMCN was also confirmed by immunofluorescence staining on HUVECs treated by 10 ng ml^−1^ TNF-α at 24 h (Fig. 3c). TNF-α as a potent proinflammatory cytokine is also known to induce the expression of the pro-adhesive molecules E-selectin, VCAM-1 and ICAM-1. Shown by flow cytometry, concomitant with the decrease of cell surface EMCN, there was an increase in pro-adhesive molecules ICAM-1, VCAM-1 and E-selectin, which reached maximal levels at 24 h (Fig. 3d). These findings demonstrate an inverse relationship between the expression of the anti-adhesive EMCN and pro-adhesive molecules under inflammatory conditions. In addition to TNF-α, treatment with IL-1β or IFN-γ also led to dose-dependent reduction of EMCN protein expression (Supplementary Fig. 1), suggesting that EMCN downregulation by inflammatory cytokines is a common feature of inflammation.

### EMCN overexpression prevents neutrophil adhesion

**Figure 4:**
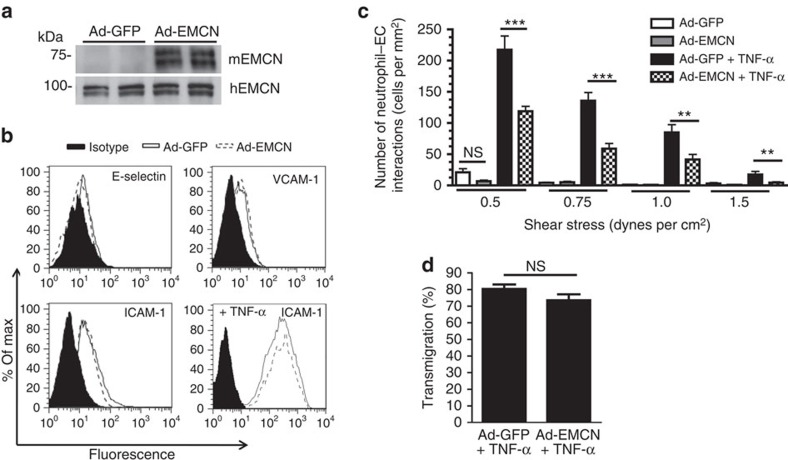
Overexpression of EMCN protects endothelial cells from neutrophil adhesion induced by treatment with TNF-α. (**a**) Sub-confluent HUVECs were transduced for 48 h with adenoviruses expressing mEMCN (Ad-EMCN) at an MOI 6. Protein biosynthesis of mouse and human EMCN (hEMCN) were determined by western blot. Transduction with GFP (Ad-GFP) at an MOI 6 was used as control. (**b**) After adenovirus transduction at MOI 6 and TNF-α stimulation (10 ng ml^−1^, 24 h), HUVECs were dissociated for evaluation of pro-adhesive molecules, E-selectin, VCAM-1 and ICAM-1, by flow cytometry. Expression of pro-adhesive molecules was unaffected by overexpression of Ad-GFP and Ad-EMCN. (**c**) Flow studies revealed that TNF-α treatment of cells overexpressing mEMCN at an MOI 6 led to a reduced total number of neutrophil–endothelial cell interactions compared to control Ad-GFP-transfected cells. (**d**) EMCN overexpression did not influence the transmigration of neutrophils that adhered to TNF-α-treated HUVEC surfaces transfected with Ad-EMCN compared to control Ad-GFP-transfected cells. Results in (**a** and **b**) represent one of three independent experiments performed. In (**c** and **d**), results are representative of three human donor experiments performed, with each independent study performed in triplicate or duplicate, respectively. Results represent mean±s.e.m. Significance was determined for (**c**) using one-way ANOVA followed by Newman–Keuls *post hoc* test or (**d**) using Student's *t*-test. **P<0.01 and ***P<0.001; NS, nonsignificant.

To further determine the extent to which EMCN can prevent neutrophil–endothelial cell interactions, murine EMCN (mEMCN) was overexpressed in HUVECs before their treatment with TNF-α for 24 h. mEMCN overexpression was accomplished by adenoviral transduction at multiplicity of infection (MOI) 6 for 48 h as demonstrated by western blot analysis (Fig. 4a). Adenoviral transduction with mEMCN (Ad-EMCN) or green fluorescent protein (Ad-GFP) did not alter the biosynthesis of pro-adhesive molecules, E-selectin, VCAM-1 and ICAM-1 (Fig. 4b). In addition, EMCN overexpression did not influence TNF-α induction of ICAM-1.

Neutrophil–endothelial cell adhesion assays were performed to determine whether mEMCN could prevent binding of neutrophils to endothelial cells, following treatment with TNF-α, which triggers high levels of pro-adhesive molecules. Analysis using the *in vitro* flow chamber assay indicated that overexpression of mEMCN significantly blocked neutrophil adhesion to stimulated HUVECs. Specifically, neutrophil adhesion to HUVECs overexpressing mEMCN decreased by up to 70% at 0.5–1.0 dynes per cm^2^ compared to control Ad-GFP-treated cells (Fig. 4c). These findings demonstrate that mEMCN is able to prevent neutrophil adhesion even after significant upregulation of pro-adhesive molecules by TNF-α. Noticeably, this suppression was achieved by overexpressing mEMCN at MOI 6, by which total EMCN protein level was comparable to the level seen in untreated control (Supplementary Fig. 2). Lower level of EMCN overexpression (at MOI 1 and 3) could not suppress TNF-α-induced neutrophil adhesion. This result suggests that normalizing EMCN protein to its physiological level is necessary for the suppression of neutrophil adhesion.

To assess whether the expression of EMCN effects neutrophil adhesion or transmigration, or both steps in stimulated HUVECs, neutrophils under flow chamber conditions were allowed to accumulate on the endothelial surface that had been treated with TNF-α (10 ng ml^−1^) for 24 h. After the designated accumulation time, the number of neutrophils transmigrating through the endothelium were determined and are represented at % transmigrated. EMCN overexpression did not prevent transmigration of adherent neutrophils as compared to Ad-GFP overexpressing HUVECs (Fig. 4d), indicating that EMCN is a barrier to neutrophil adhesion, and does not influence cell transmigration.

### Role of EMCN in inflammation *in vivo*

To assess the contribution of EMCN to neutrophil–endothelial cell interactions *in vivo*, we injected the potent proinflammatory cytokine TNF-α into the vitreous cavity of the eye to induce the influx of leukocytes. As a negative control, mice received a similar injection of saline. To determine whether infiltration of leukocytes cells can be altered by overexpression of EMCN, 7 days before the TNF-α injection, mice received an intravitreal injection of either Ad-EMCN, or Ad-GFP as a negative control.

**Figure 5:**
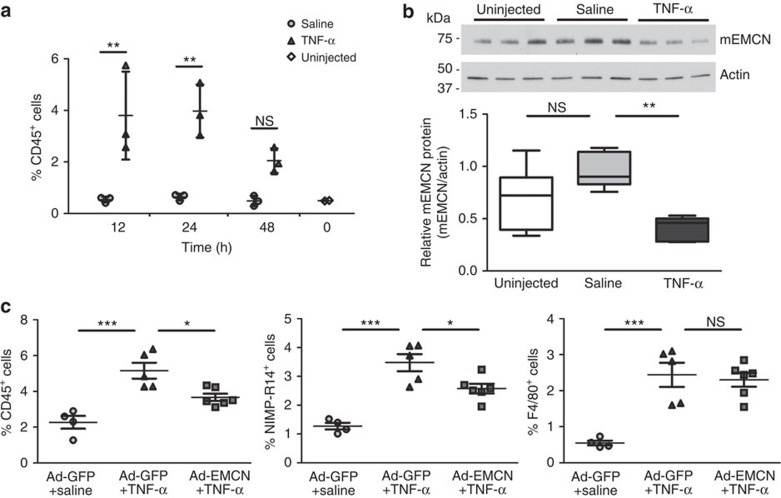
Overexpression of EMCN *in vivo* protects against TNF-α-induced inflammatory cell infiltrations. (**a**) Ciliary bodies were collected 12, 24 and 48 h post intravitreal injection of saline or TNF-α (10 ng per 1 μl), as well as uninjected controls. Following cell dissociation from ciliary bodies, CD45^+^ cells were stained and analysed by flow cytometry. TNF-α led to a significant increase of CD45^+^ cells at 12 and 24 h when compared to saline controls. Each sample was pooled from two eyes and three samples were evaluated for saline or TNF-α-injected group. (**b**) Ciliary bodies were collected from eyes 24 h after intravitreal injection of TNF-α (10 ng per 1 μl), saline or uninjected. Tissue lysates were processed for western blot, and analysed by ImageJ software. TNF-α led to a significant reduction of EMCN protein in ciliary bodies compared to saline-injected controls. Further, saline injections did not significantly change the levels of EMCN protein compared to uninjected controls. Each sample was pooled from two eyes and six samples were evaluated for each group. The box and whisker plot show the 25 and 75 percentiles (box), the median and the minimum and maximum data values (whiskers). (**c**) Eyes were injected intravitreally with Ad-EMCN or Ad-GFP, followed by TNF-α (10 ng per 1 μl) or saline 7 days later. Ciliary bodies were collected 24 h post TNF-α injection, dissociated, stained for CD45, NIMP-R14 and F4/80 and analysed by flow cytometry. TNF-α led to significant increases of CD45^+^, NIMP-R14^+^ and F4/80^+^ cells compared to saline-injected group with Ad-GFP injection; Ad-EMCN significantly suppressed TNF-α-induced increase in CD45^+^ and NIMP-R14^+^ cells compared to Ad-GFP-injected group. Each sample was pooled from two eyes and four to six samples were evaluated for each group. Results are displayed as mean±s.e.m. Significance was determined for (**a**) using two-way ANOVA with Bonferroni's *post hoc* test or (**b** and **c**) using one-way ANOVA with Tukey's *post hoc* test. *P<0.05, **P<0.01 and ***P<0.001; NS, nonsignificant.

As the primary site of leukocyte infiltration in the eye is the ciliary body^24^, we first investigated the time course of TNF-α-induced inflammatory cell infiltration in ciliary body vessels *in situ*. Flow cytometry was conducted on dissociated ciliary body cells stained for CD45 (a pan-leukocyte marker) from eyes collected 12, 24 and 48 h after TNF-α injection (Fig. 5a). At 12 and 24 h, TNF-α treatment led to ∼7-fold increase of CD45^+^ cells compared to saline-injected controls at the same time points. At 48 h, there was still a trend of increase in CD45^+^ cells but at a lower level compared to 12 and 24 h. Second, the effects of TNF-α on EMCN expression in ciliary body vessels were evaluated by western blot (Fig. 5b; Supplementary Fig. 3). Consistent with our *in vitro* findings, 24 h TNF-α treatment reduced the level of ciliary body EMCN by ∼50% as compared to saline-injected controls (Fig. 5b). Although the reduction of EMCN in ciliary body by TNF-α was persistent at 48 h (Supplementary Fig. 3), the effect of EMCN overexpression on inflammatory cell infiltration was evaluated at 24 h given that the increase of CD45^+^ cells peaks at 24 h.

Ad-EMCN or Ad-GFP as control was injected intravitreally 7 days before TNF-α injection. Adenoviral transduction of the ciliary body vessels was confirmed by co-localized expression of GFP and endothelial cell marker using confocal microscopy (Supplementary Fig. 4a), and by quantification of CD31^+^GFP^+^ cells using flow cytometry (Supplementary Fig. 4b). Two inflammatory cell markers (NIMP-R14 for neutrophil and F4/80 for monocyte/macrophage) were used in addition to CD45 to evaluate inflammatory cell infiltration. First, it was confirmed that TNF-α led to significant increases of NIMP-R14^+^, F4/80^+^ and CD45^+^ cells in ciliary body 24 h post TNF-α injection compared to saline-injected controls within the Ad-GFP groups (Fig. 5c). With Ad-EMCN injection, however, TNF-α-triggered increases in CD45^+^ cells and NIMP-R14^+^ cells were reduced by ∼50 and ∼40%, respectively, compared to the group that received both Ad-GFP and TNF-α, whereas there was no significant change in F4/80^+^ cells (Fig. 5c).

## Discussion

Under normal, non-inflammatory conditions, the vascular endothelium maintains an apical surface in which leukocytes and platelets do not adhere. Once inflammation is initiated, leukocyte trafficking is coordinated by sequential adhesive interactions between the blood leukocytes and endothelial cells^25^. The prevailing paradigm is that circulating leukocytes localize specifically at the inflammation site due to proinflammatory cytokines that are produced and released by interstitial or paracellular resident leukocytes, which leads to endothelial cell activation. The endothelial glycocalyx is the primary site of the rapid transition between non-adherent and adherent states, and a vast array of adhesive molecules both on leukocytes and endothelial cells has now been described^6^,^8^,^26^. The non-inflammatory nature of the endothelial surface has been largely ascribed to the absence of pro-adhesive receptors and the possible presence of a functional anti-adhesive molecule has not been investigated. The endothelium plays a major role in regulating adhesiveness by providing a strong negative charge^12^ and exposing membrane proteins with glycosylation motifs that have both anti-adhesive and pro-adhesive roles, such as members of the CD34 family^18^. Here we have documented the biological importance of an endothelial-specific sialomucin, EMCN, in maintaining an anti-inflammatory endothelial cell surface.

Although previous studies^22^,^27^ have suggested an anti-adhesive function for EMCN, our data reveals the biological importance of EMCN localization and expression in the vascular endothelium under normal and inflammatory conditions. siRNA knockdown of EMCN led to a significant increase in neutrophil–endothelial cell adhesion under a range of physiologically relevant laminar shear stresses. Flow cytometry analysis of HUVECs treated with siRNA targeting EMCN demonstrated that this effect was due to the depletion of EMCN and not to an increase in expression of pro-adhesive molecules, as E-selectin, VCAM-1 and ICAM-1 were not by affected by the depletion of EMCN. ICAM-1, which is involved in leukocyte firm adhesion during inflammation, is constitutively expressed by HUVECs^1^,^11^ and was unchanged by siRNA against EMCN. In light of the known relative sizes and conformation of EMCN (75–90 kDa; extended mucin-like ectodomain) and ICAM-1 (90 kDa; multiple extracellular Ig-like loops)^28^, we speculated that depletion of EMCN was unmasking ICAM-1, and allowing neutrophil adhesion. The ability of neutralizing mAb against LFA-1 on neutrophils to disrupt neutrophil binding to ICAM-1 on HUVECs supported this hypothesis and also demonstrated that the EMCN in the endothelial cell glycocalyx, possibly by exposing negatively charged *O*-glycans that provide charge repulsion and steric hindrance, is critical to maintaining an anti-adhesive surface under non-inflammatory conditions. Interestingly, *in vivo* studies have shown EMCN localization to the luminal surface of venous and capillary, but not arterial, endothelium^19^,^20^. *In vitro*, however, EMCN expression has been observed in human aortic and microvascular endothelial cells^29^. These data suggest that laminar shear stress may play an important role in regulating EMCN expression, since expression of EMCN in the cell lines was determined under static conditions, whereas the reported absence of EMCN in arterial endothelium was in tissue where shear stress is present. This hypothesis is supported by data indicating that prolonged exposure to high shear stress downregulates expression and surface localization of EMCN in HUVECs (Supplementary Fig. 5). Taken together, our observations are consistent with the idea that the glycocalyx provides a first line of defence against leukocyte adhesion and furthermore, support a role for EMCN in limiting access of leukocytes to cell surface adhesion molecules.

EMCN has been shown to include the MECA-79 carbohydrate epitope that is important in L-selectin-mediated interactions^21^. Previous work has suggested that EMCN supports L-selectin-mediated rolling, and is thus a pro-adhesive molecule^30^. However, the data to support this conclusion were generated using CHO cells overexpressing EMCN along with a specific combination of carbohydrate modifying enzymes, glycosyltransferases and sulfotransferases, which generates L-selectin-reactive capping group in core-2 branched *O*-glycans attached to sialomucin core proteins, such as EMCN. Thus, this experimental system that was used to demonstrate that EMCN supports L-selectin-mediated rolling along glass capillaries coated with a neutralizing anti-L-selectin mAb appears unlikely to be relevant to the endothelium *in vivo*.

Given the critical role of EMCN in maintaining a non-inflammatory phenotype, we investigated its expression under inflammatory conditions. A primary focus in the study of inflammation has been on the promotion of leukocyte recruitment and extravasation, and much less is known about the potential contribution of anti-adhesive molecules. Previous studies have shown that components of the glycocalyx, including heparan sulfate and chondroitin sulfate, are shed in response to injury or cytokine-mediated inflammation^13^,^14^,^15^. Thus, we speculated that EMCN levels would be reduced in response to TNF-α. In agreement with this notion, we found that TNF-α treatment of HUVECs led to a decrease in EMCN mRNA and surface protein, and an increase in the pro-adhesive molecules E-selectin, VCAM-1 and ICAM-1. A potential mechanism of EMCN downregulation by TNF-α is likely to involve binding to the membrane receptors TNF-R55 and TNF-R75. TNF-R55 is primarily responsible for the stimulation of leukocyte adhesion to the endothelium by upregulation of adhesion molecules on the endothelial cell surface^31^, but the role of these membrane receptors in the downregulation of EMCN has not been yet examined. TNF-α is also known to control the activation of GATA-2 (ref. 32), a key transcription factor commonly expressed in endothelial cells. Since GATA-2 elements are present in EMCN gene regulatory sequences^33^, it is tempting to speculate a potential role for GATA-2 in modulating EMCN expression under proinflammatory conditions. Contrary to our results, a previous study has reported increased EMCN expression upon TNF-α treatment^29^. It is difficult, however, to draw conclusions from this study, since results were shown in sparse culture conditions, which demonstrated significant variability in the basal level of EMCN expression, protein levels were not shown, and the authors did not determine the statistical significance of the data.

Motivated by the concept that the endothelial cell glycocalyx has the capacity to prevent leukocyte adhesion to constitutively expressed ICAM-1, we assessed the ability of EMCN to interfere with the pro-adhesive effects of TNF-α. To accomplish this, we treated HUVECs that were overexpressing EMCN with TNF-α and examined neutrophil adhesion. Remarkably, overexpression of EMCN prevented neutrophil adhesion to TNF-α stimulated HUVECs under a range of physiologically relevant shear stresses.

Given the accessibility of the eye for study and the fact that several ocular pathologies, including uveitis^34^,^35^, diabetic retinopathy^36^,^37^ and glaucoma^35^, have inflammatory components, we generated ocular inflammation to assess the role of EMCN *in vivo*, and examined the level of EMCN protein in the ciliary body, the primary site of neutrophil infiltration during intraocular inflammation. Consistent with our *in vitro* observations, intravitreal injection of TNF-α led to decreased of EMCN in the ciliary body within 24 h of treatment. Importantly, overexpression of EMCN dramatically reduced the accumulation of CD45^+^ cells as well as neutrophils (NIMP-R14^+^ cells) in the ciliary body following TNF-α injection. On the basis of the results in this experimental model, it is tempting to speculate that promoting expression of EMCN to physiological levels would prevent neutrophil extravasation into inflamed tissue, but this question still remains unanswered *in vivo*. Overall, our observations introduce the novel paradigm that the downregulation of anti-adhesive molecules may be as important in transforming the endothelial cell surface to a proinflammatory state as the elevation in adhesive molecules. Moreover, our *in vivo* results provide an exciting new therapeutic option for the manipulation of the inflammatory process.

## Methods

### Mice

C57BL/6 female mice, 6–8 week in age, were purchased from Jackson Laboratories (Bar Harbor, Maine). Mice were handled in accordance to the National Institute of Health Guide for the Care and Use of Laboratory Animals. All procedures were approved by the Institutional Animal Care and Use Committee of the Schepens Eye Research Institute/Mass Eye and Ear.

### Reagents and antibodies

Recombinant human TNF-α was purchased from Pepro Tech. Blocking mAb against β2 integrin (TS1/18) and MHC class I mAb (W6/32) were purchased from American Type Culture Collection (ATCC). The following primary antibodies were used: rat anti-human EMCN (1:1,000, ab45771, Abcam), rat anti-mouse EMCN V.7C7 (1:1,000, sc-65495, Santa Cruz Biotechnology), mouse anti-actin (1:1,000, Santa Cruz Biotechnology), mouse anti-tubulin (1:2,000, CalBiochem) and rabbit anti-GAPDH (1:1,000, Santa Cruz Biotechnology). Antisera against human E-selectin (clone CL2), VCAM-1 (clone E1/6) and ICAM-1 (clone Hu 5/3) were purchased from ATCC. CD31 was detected using rat anti-mouse CD31 (1:250, BD Pharmingen). For flow cytometry of cells from ciliary body, PE-NIMP-R14 (Novus), Brilliant Violet 421 anti-CD45 and PE-Cy7 anti-F4/80 (Biolegend) and PE-CD31 (BD Pharmingen) or their corresponding isotype controls were used.

### Cell culture

Primary HUVECs (provided by the Center for Excellence in Vascular Biology, Brigham and Women's Hospital, Boston, MA) were cultured on 0.1% gelatin coated T-75 flasks in EGM-2 medium (EBM-2, SingleQuots including VEGF, Lonza) supplemented with 20% fetal bovine serum (FBS), L-glutamine, and penicillin/streptomycin. For all experiments, HUVECs between passage 1 and 4 were used. Where indicated, confluent HUVECs were treated with TNF-α (10 and 25 ng ml^−1^) for 4 and 24 h. Cells were cultured for 12 h in medium (EBM-2, 0.5% FBS, 25 mM HEPES, penicillin/streptomycin) prior being subject to TNF-α treatments.

### Leukocyte isolation

The leukocyte isolation protocol used in this study was approved by Brigham and Women's Institutional Review Board. Informed consent was obtained from all volunteers. Blood was drawn from healthy adult donors. Neutrophils were prepared from heparinized peripheral blood by density sedimentation in a discontinuous gradient of Ficoll and Histopaque-1119. Care was taken to minimize physical disruption or temperature changes to the neutrophil preparation. Neutrophils were suspended at 5 × 10^5^ cells per ml in Dulbecco's PBS containing 0.2% human serum albumin.

### Flow apparatus design and application

The parallel-plate flow chamber used in the present study has been described previously in detail^38^. Briefly, the flow chamber consists of two stainless steel plates separated by a silastic gasket. The lower plate has a circular cutout where a glass coverslip coated with an endothelial cell monolayer is placed. Fluid is then drawn through the chamber at defined flow rates using a syringe pump. In our experiments, HUVEC monolayers were grown on 25-mm glass coverslips coated with 5 μg ml^−1^ fibronectin (Sigma). In TNF-α treated samples, HUVEC monolayers were incubated with 10 ng ml^−1^ TNF-α for 24 h before the assay was performed. The coverslip was positioned into the flow chamber and the entire chamber was mounted on an inverted microscope. Neutrophils were drawn through the chamber at an estimated shear stress of 1.5 dynes per cm^2^ for 2 min, and then decreased by 0.5 dynes per cm^2^ every 2 min to 0.5 dynes per cm^2^. Cells were pre-exposed to each laminar shear stress condition for 1 min before recording neutrophil adhesion for another min. Interactions were recorded with a phase-contrast objective (× 20), and a video microscope connected to Videolab software (Ed Marcus Laboratories, Boston, MA). The number of neutrophil–EC interactions, including rolling and firmly adhered neutrophils, was counted in six different fields after an initial min at each flow rate^39^,^40^. For blocking experiments, neutrophils, 0.5 × 10^6^ cells per ml were incubated with mAbs (TS1/18, W6/32), at 20 μg ml^−1^ for 30 min at 4 °C.

### Neutrophil transmigration

The live-cell fluorescence microscopy flow model has been described previously^41^. HUVEC monolayers on coverslips were activated with TNF-α (10 ng ml^−1^, 24 h) and inserted into the flow chamber. Neutrophils (1 × 10^6^ per ml) were drawn across HUVECs at 1.0 dynes per cm^2^ and adhesion and transmigration monitored by time-lapse, live-cell microscopy performed on a Nikon-TE-200 microscope equipped with a × 20 objective and a heating stage maintained at 37 °C. Differential interference contrast (DIC) optics was used to visualize neutrophil transmigration.

### Western blotting

Cells in culture were collected and lysed in RIPA buffer (Sigma, USA) containing protease (Complete, Mini, EDTA-free; Roche) and phosphatase inhibitors (phosphatase inhibitor cocktails 2, Sigma) at 4 °C for 5 min. Protein concentrations were determined using the BCA protein assay kit (Pierce) after three cycles of freeze thaw. For protein collected from tissues, the ciliary bodies from two eyes were pooled and disrupted in lysis buffer (Cell Signaling Technology, MA) containing protease (Complete, Mini, EDTA-free; Roche) and phosphatase inhibitors (phosphatase inhibitor cocktails 2, Sigma, USA), and exposed to high frequency ultra-sonication. Equal amounts of protein (10–50 μg for cell lysate, and 3–5 μg for tissue lysate) were separated by SDS–polyacrylamide gel electrophoresis (10% acrylamide) under reducing conditions and transferred to PVDF membranes (Immobilon-P, Millipore). Membranes were blocked in 1 × TBS containing 0.1% Tween, 5% non-fat milk and 2.5% BSA for 1 h, and then probed with primary antibody overnight at 4 °C. Secondary antibody incubations were performed with the corresponding horseradish peroxidase-conjugated antibody at room temperature for 1 h. Proteins were detected by chemiluminescence with SuperSignal West Pico or Dura (Thermo Scientific).

### Biotinylation of cell surface proteins

To determine the presence and relative protein levels of EMCN on the endothelial cell membrane, cell surface proteins were biotinylated and isolated by chromatography on a NeutrAvidin-agarose affinity column using Pinpoint cell surface protein isolation kit (Pierce, Rockford, IL), according to the manufacturer's instructions. Membrane extracts of the labelled cells were analysed for the presence of EMCN by western blot, as described above.

### RNA isolation and real-time PCR analysis

RNA was collected from HUVECs in TRIzol and extracted according to manufacturer's instructions. DNase treatment was performed using DNase 1 from Ambion (cat# AM2224). First-strand cDNA was synthesized from 1 μg of total RNA with oligo(dT)_20_ primer and reverse transcriptase (iScript, Bio-Rad Laboratories, USA) in a 25-μl reaction, following the manufacturer's protocol. For real-time PCR, reactions were performed on the ABI Prism 97000 Sequence Detection System (Applied Biosystems) using 5 μl of cDNA, 1 μl of 10 mM forward and reverse primer, 12.5 μl SYBR Green PCR master mix (Applied Biosystems) and 5.5 μl of H_2_O, according to the manufacturer's instructions. Amplification of GAPDH was performed on each sample as a control for normalization.

EMCN: forward (5′-ATTTGTTCTGGTGGGTTTGT-3′)

Reverse (5′-TGCAGGACTTTCTCCTTTTC-3′)

GAPDH: forward (5′-CCCATCACCATCTTCCAGGA-3′)

Reverse (5′-CATCGCCCCACTTGATTTTG-3′)

### siRNA knockdown

HUVECs were seeded at 50% confluence 1 day before transfection. Cells were washed with 1 × PBS to remove residual antibiotics and equilibrated in OPTI-MEM. siRNA against EMCN (siRNA EMCN #3 AS00KOXP, cat# 4399665, ID S229102) or the control siRNA (Ambion: 50 mM stock solution) were incubated with Oligofectamine (Invitrogen) 1:4 ratio in OPTI-MEM at room temperature for 20 min to form complexes, then added to cells at a final siRNA concentration of 200 nmol ml^−1^. After 6 h, complete growth medium without antibiotics and VEGF was added to the cells. After 24 h, cells were placed into normal growth medium. Proteins were collected after 48 h of transfection.

### Histological analysis and microscopy

Tissues were dissected from adult mice that were anesthetized with ketamine/xylazine and PBS-perfused via the aorta. After overnight fixation at 4 °C with 4% paraformaldehyde in PBS, tissues were washed with PBS, and cryoprotected in 20% sucrose. Tissues were then infiltrated overnight at 4 °C and embedded the next day with the same 2:1 mixture of 20% sucrose and O.C.T. compound before being frozen on dry ice.

For identification of vascular cell type, tissue sections were subjected to immunoperoxidase staining. After washing with PBS and permeabilization with PBS containing 0.2% Tween 20 (PBST), endogenous peroxidase activity was blocked by incubation with 1% H_2_O_2_ in PBS for 10 min. Non-specific binding was blocked by incubation with blocking buffer (3% rabbit serum in PBST) for 30 min at room temperature. Tissue sections were incubated with either rat anti-mouse CD31 (1:250, BD Pharmingen) or rat anti-mouse EMCN (1:1,000, mAb V.7C7, Santa Cruz) diluted in blocking buffer overnight at 4 °C. A section incubated with rat IgG as a negative control was included in each experiment. Slides were examined and images were captured using a Zeiss Axioskop 2 MOT microscope with a mounted AxioCam digital camera under both × 20 and × 40 objective lenses. Post-acquisition processing of images was conducted using Adobe Photoshop.

### Immunofluorescence

A biopsy of healthy human skin of a 72-year-old female was obtained with informed consent, following an approval of the Human Studies Committee at Schepens Eye Research Institute/Massachusetts Eye and Ear. The biopsy was fixed in 4% paraformaldehyde in PBS at room temperature for 24 h. The tissue was embedded in paraffin, sectioned and processed by standard techniques. The rehydrated tissue sections were treated with Vector-Antigen UnMasking solution for 20 min followed by pretreatment with sodium borohydrate 0.1% in PBS for 5 min. The sections were then washed in 1 × PBS and blocked for 1 h at room temperature in 3% goat serum, 3% donkey serum, 2.5% BSA, 0.2% Tween 20. Immunofluorescent staining for EMCN and collagen IV was performed using monoclonal rat anti-human EMCN (1:1,000, Abcam) and mouse anti-human collagen-IV (1:500, Sigma) overnight at 4 °C. Secondary DyLight 550-conjugated goat anti-rat (1:300, Abcam) and Alexa Fluor 488-conjugated goat anti-mouse (1:300, Invitrogen) antibodies, and 4,6-diamidino-2-phenylindole (DAPI) for cell nucleus, were used and incubated for 45 min at room temperature. For each experiment, a section was incubated with isotype-matched IgG as a negative control.

For localization of EMCN in cell culture, HUVECs grown on fibronectin treated glass coverslips were fixed in 1% formalin at 4 °C overnight, permeabilized with 0.01% Triton X-100 in PBS, washed three times for 5 min in 1 × PBS, and blocked with 3% secondary serum species, 1% BSA for 1 h at room temperature. Coverslips were then incubated in rat anti-human EMCN Ab (1:500, Abcam) at 4 °C overnight, followed by 3 × 5 min washes in 1 × PBS. The cells were then incubated in the secondary fluorescent antibodies (DyLight 550) for 1 h at room temperature and washed in 1 × PBS. Fluorescent images were taken with Zeiss Fluorescent Microscope (Axioskop 2 MOT Plus, × 40 objective). Analysis of EMCN fluorescence intensity was performed using ImageJ software (National Institutes of Health, Bethesda, MD). Fluorescence intensity was measured in two different spots of each endothelial cell in 40 different cells in images corresponding to two different experiments and two different fields of view. Values were normalized by dividing each by the average value corresponding to EMCN fluorescence intensity in control cells.

### Flow cytometry

For cultured cells, HUVECs were detached non-enzymatically using cell-dissociation buffer (Gibco), collected by centrifugation 1,000*g* for 5 min, washed two times with cold 1 × PBS and fixed in 1% formaldehyde for 30 min at room temperature, followed by three washes in 1 × PBS. Cells (1 × 10^6^ per ml) were treated with the indicated mAb to ICAM-1, VCAM-1 and E-selectin (1:50, ATCC) for 20 min on ice, followed by incubation with fluorescein isothiocyanate or phycoerythrin (PE)-conjugated goat anti-mouse antibody (1:50) on ice for 30 min. Cell surface levels were analysed using a FACSCalibur (Becton Dickinson). Histograms of per cent max versus fluorescence (FL1-4) were plotted on the *y* and *x* axis, respectively.

For ciliary body tissue, samples were digested with collagenase IV (1,000 U ml^−1^, Worthington) and DNase I (1 U ml^−1^, Sigma) in PBS at 37 °C for 1 h. Cells were then dissociated by gentle pipetting and collected after filtering through 70 μm cell strainers (BD). After wash, cells were divided equally into two tubes for each antibody and its isotype control, or into three tubes to include unstained control. Cells were blocked with 1% BSA in PBS for 10 min and stained with directly conjugated antibodies: PE-NIMP-R14 (Novus), Brilliant Violet 421 anti-CD45 and PE-Cy7 anti-F4/80 (Biolegend) or their corresponding isotype controls for 30 min at 4 °C. After wash, cells were resuspended in 500 μl 1% BSA in PBS, and cell fluorescence was measured by using a BD LSR II flow cytometer. Data were acquired until 200,000 events were collected. Cells from the Ad-GFP^+^TNF-α group were stained with each antibody and its isotype control, then analysed by flow cytometry to set up the compensation. Data analysis was performed using FlowJo, and results represented as the percentage of positive cells after compensation and correction using isotype controls.

### EMCN adenovirus production and transduction

A cDNA encoding full-length mEMCN (provided by Dietmar Vestweber, Division of Vascular Cell Biology, Max-Planck-Institute, Germany) was cloned into a pShuttle vector using standard molecular biology techniques. Clones containing the EMCN cDNA were amplified in DH5α cells, linearized and dephosphorylated. The linearized plasmid was co-transformed with the pAdEasy vector (Stratagene) into BJ5183 cells to produce recombinant adenoviral plasmid through homologous recombination. Enhanced GFP (EGFP) was similarly cloned into pAdEasy as a control. The engineered adenoviral vectors were amplified in DH5α cells and purified using Maxiprep plasmid purification columns (Qiagen). Purified adenoviral plasmids were linearized and transfected into 293A cells. Virus was amplified from a single plaque by serial transduction onto 15 cm^2^ plates of 293A cells and purified by two cycles of CsCl gradients. Purified virus was verified by restriction digest and sequence analysis of viral DNA from both preparations. The final products were tittered by optical absorbance methods. The result was converted and expressed as plaque forming units (p.f.u.) per ml. The EMCN adenovirus titre was 3.1 × 10^10^ p.f.u. ml^−1^ and EGFP adenovirus titre 1 × 10^11^ p.f.u. ml^−1^. The virus was distributed in the following formulation: 50 mM Tris-HCl (pH 7.4), 5 mM EDTA, 1.4 M CsCl, 50 mM NaCl, 0.5 mM MgCl_2_ and 25% glycerol. Virus production, purification, verification and tittering were performed by the Harvard Gene Therapy Initiative, Boston, MA. HUVECs were seeded at 50% confluence 1 day before adenoviral transduction. Sub-confluent cells were transduced with Ad-GFP or Ad-EMCN at an MOI 6 for 48 h in normal growth medium, cells were thereafter washed with 1 × PBS and processed for protein. For the experiments in which cells were stimulated with TNF-α, the cells were first transduced with the adenoviruses for 24 h and then treated with TNF-α (10 ng ml^−1^) and processed for protein 24 h later.

### Intravitreal injections

Mice were anesthetized by intraperitoneal injection of a mixture of ketamine–xylazine (100 mg kg^−1^ ketamine/20 mg kg^−1^ xylazine body weight). By using a 30-G hypodermic needle, an incision was made just posterior to the limbal vessels. A coverslip was used to applanate the cornea to allow view to the posterior pole of the eye. Injections were performed using a Hamilton syringe fitted with a 33-G needle placed directly above the optic nerve head. Mice received an intravitreal injection of TNF-α (Millipore/Chemicon, 10 ng in 1 μl saline) or as a negative control, 1 μl injection of saline only. Ciliary bodies from two eyes were pooled and collected at designated time points post TNF-α injection to determine EMCN protein levels by western blot analysis or inflammatory cell infiltration by flow cytometry. Overexpression of EMCN was achieved by intravitreal injection of Ad-EMCN (3.1 × 10^7^ p.f.u. μl^−1^) 7 days before TNF-α injection, with Ad-GFP injection (3.1 × 10^7^ p.f.u. μl^−1^) as controls. Slit lamp examination was conducted before TNF-α injection, and mice that displayed signs of inflammation (clouding of the anterior or posterior chamber) were excluded from the study.

### Statistics

Statistical analysis was carried out with Prism 4.0b for Macintosh. All data are shown as mean±s.e.m. The mean values from each group were compared by Student's *t*-test, one-way analysis of variance (ANOVA) with Tukey's *post hoc* test, one-way ANOVA followed by Newman–Keuls *post hoc* test or two-way ANOVA with Bonferroni's *post hoc* test. In all tests, *P* values <0.05 were considered statistically significant.

## Additional information

**How to cite this article:** Zahr, A. *et al.* Endomucin prevents leukocyte–endothelial cell adhesion and has a critical role under resting and inflammatory conditions. *Nat. Commun.* 7:10363 doi: 10.1038/ncomms10363 (2016).

## Supplementary Material

Supplementary Information Supplementary Figures 1-6
