# Supplementary material for: Endomucin prevents leukocyte–endothelial cell adhesion and has a critical role under resting and inflammatory conditions
Source: Nat Commun. 2016 Feb 2;7:10363. doi: 10.1038/ncomms10363 (PMC4740757; doi:10.1038/ncomms10363)
Supplement: Supplementary Information — Supplementary Figures 1-6 [file ncomms10363-s1.pdf]

Supplementary Figure 1

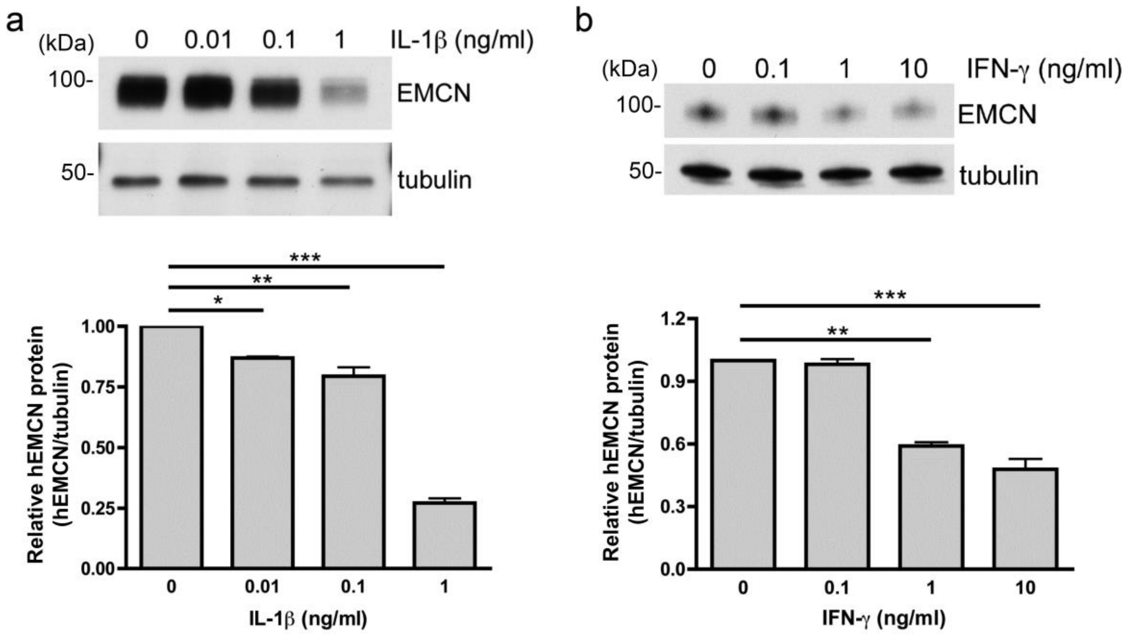

Supplementary Figure 1. IL-1β and IFN-γ treatment leads to dose-dependent reduction in EMCN total protein levels. Total cell lysates were harvested from HUVEC following 24 hr treatment with IL-1β (0.01-1 ng/ml) or IFN-γ (0.1-10 ng/ml). EMCN levels were evaluated by western blot. A dose-dependent reduction of EMCN protein was observed with IL-1β **(a)** and IFN-γ **(b)**. Results are displayed as mean ± SEM (N=3). Significance was determined using one-way ANOVA with Tukey's post-hoc test. *p<0.05, **p<0.01, ***p<0.001.

Supplementary Figure 2

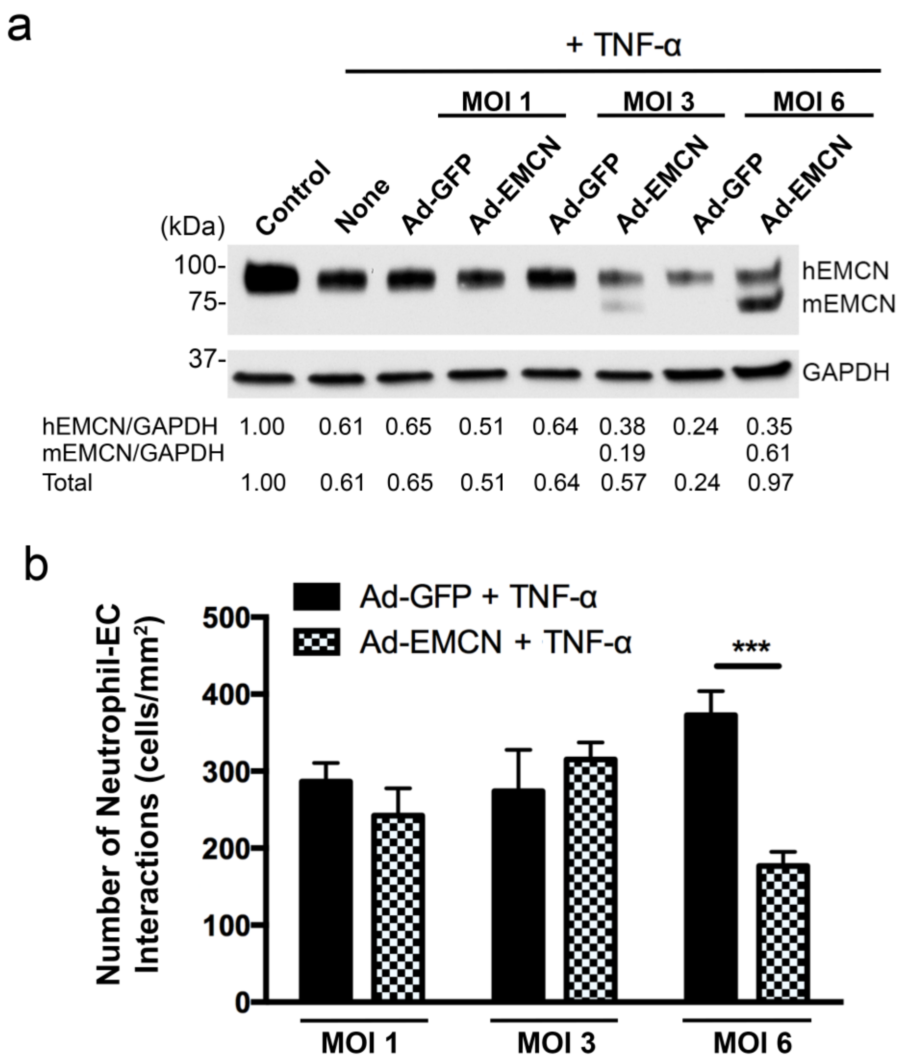

Supplementary Figure 2. Overexpression of EMCN at MOI 6 suppresses TNF-α-induced neutrophil interactions. HUVEC were transduced with Ad-GFP or Ad-EMCN using different MOI (0, 1, 3, 6) and treated with TNF-α. **(a)** Total cell lysates were harvested 24 hr after TNF-α treatment. Protein levels of endogenous hEMCN, mEMCN, and GAPDH loading control were determined by western blot and analyzed using ImageJ. This representative blot shows dose-dependent overexpression of mEMCN with increased MOI. The level of total EMCN, including hEMCN and mEMCN, at MOI 6 is comparable to untreated control. **(b)** Flow adhesion assay was performed on HUVEC from parallel plates using freshly isolated neutrophils under 0.5 dynes/cm^2 shear stress. At MOI 6 but not at MOI 1 and 3, Ad-EMCN suppressed TNF-α-induced neutrophil interactions compared to Ad-GFP control at the same MOI. Data in **(b)** are expressed as mean ± SEM. Significance was determined using one-way ANOVA followed by Newman–Keuls post-hoc test. ***p<0.001.

Supplementary Figure 3

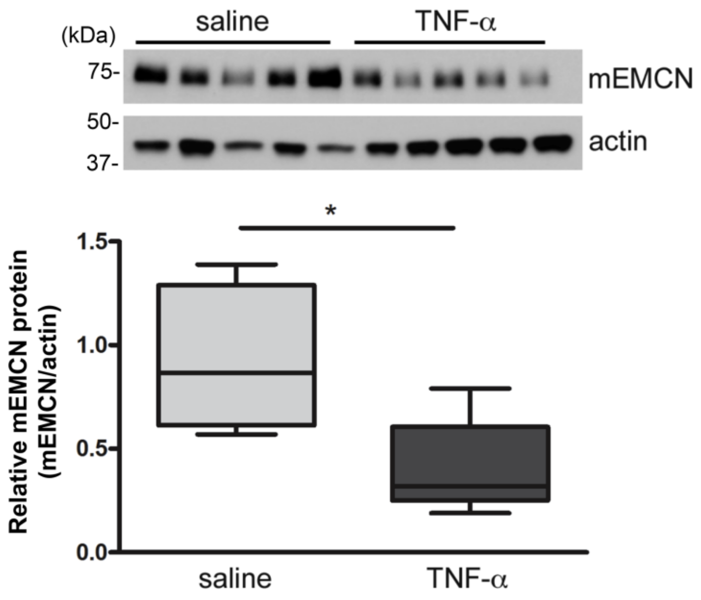

Supplementary Figure 3. Reduction of EMCN expression in the ciliary body 48 hr following TNF-α injection. Ciliary bodies were harvested 48 hr after intravitreal injection of TNF-α (10 ng/1µl) for western blot analysis of mEMCN and actin (as loading control). TNF-α led to significant reduction of EMCN in the ciliary body compared to saline-injected controls. Results are displayed as mean ± SEM (N=4-5). Significance was determined using the Student's t-test. *$p<0.05$.

Supplementary Figure 4

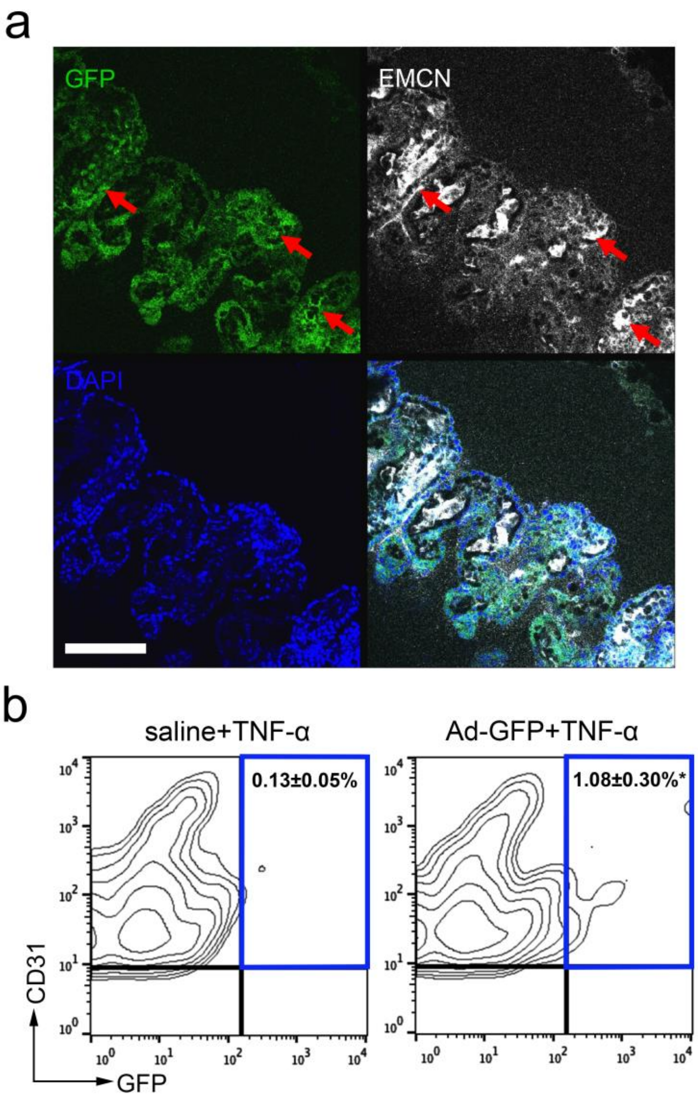

Supplementary Figure 4. Intravitreal injection allows adenoviral delivery in the mouse ciliary body. (a) Anterior segments of the mouse eye, containing the ciliary body, were harvested 8 days post Ad-GFP injection, flat mounted onto a coverglass, and fixed in 4% PFA. Confocal microscopy images show the ciliary body stained with vascular endothelial cell marker (EMCN) and GFP-expressing endothelial cells, denoted by red arrows. The scale bar represents 100 μm. **(b)** Mice received an intravitreal injection of Ad-GFP or saline injection, and 7 days later, a second intravitreal injection of TNF-α (10 ng/1μl). After 24 hr, cells from the ciliary body were harvested for CD31 staining and flow cytometry analysis. An initial gate was used to identify all CD31+ cells in the CB; the blue gate displays double positive cells within the CD31+ population. A representative contour plot is shown, with the frequency of double positive cells displayed as mean ± SEM (N=5-6). There was a significant increase in the percentage of GFP+ cells in the endothelium of CB from Ad-GFP-injected eyes compared to saline-injected controls. Significance was determined using the Student’s t-test. *$p<0.05$.

Supplementary Figure 5

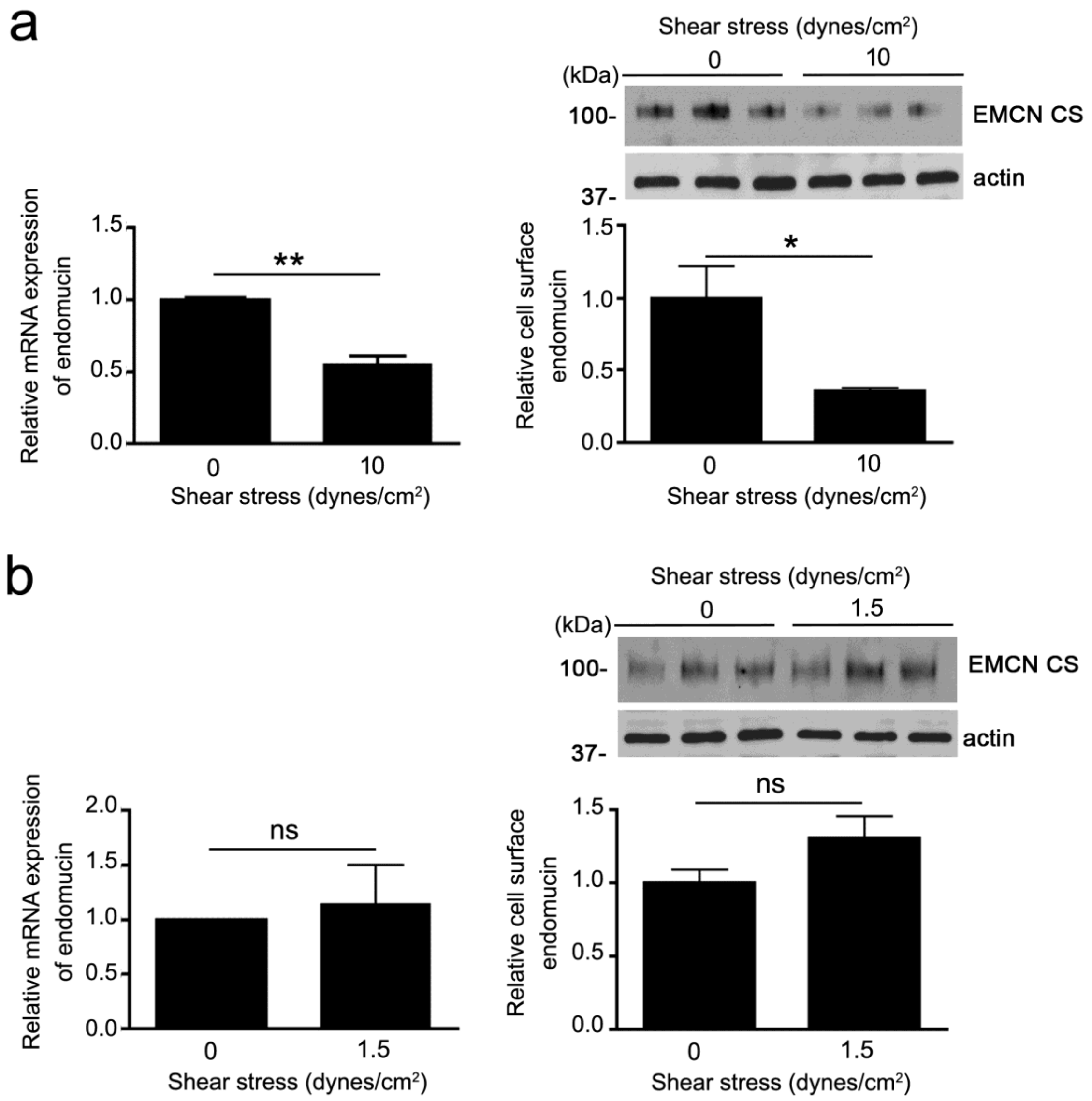

Supplementary Figure 5. High shear stress downregulates expression and surface localization of EMCN in HUVEC. HUVEC were placed on an orbital shaker and exposed to shear stress for 24 hr. **(a)** High shear stress (10 dynes/cm^2) downregulated EMCN compared to static conditions (0 dynes/cm^2) as determined by qRT-PCR and western blot analysis of biotinylated proteins. **(b)** Venular-like shear stress of 1.5 dynes/cm^2 had no effect on EMCN mRNA and cell surface protein levels. Results are displayed as mean ± SEM (N=3). Significance was determined using the Student's t-test. *p<0.05; **p<0.01. ns, non-significant. CS, cell surface.

Supplementary Figure 6

Fig. 2a

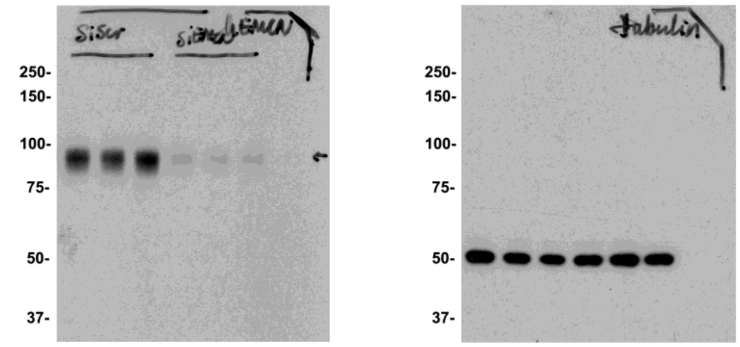

EMCN Tubulin

Fig. 3b

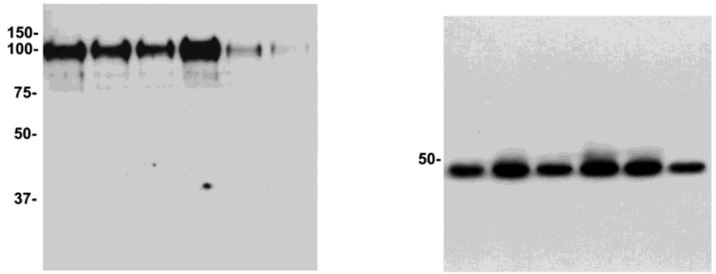

EMCN CS Actin

Fig. 4a

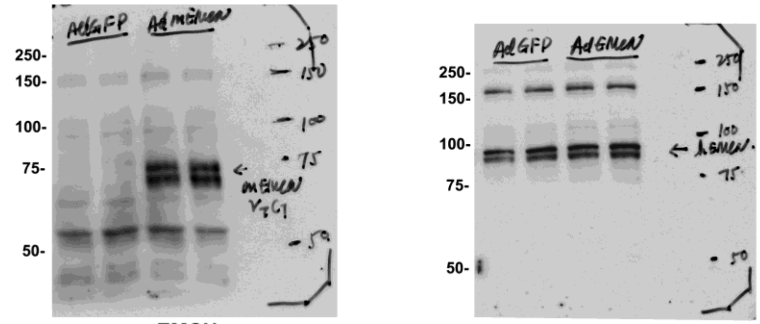

mEMCN hEMCN

Fig. 5b

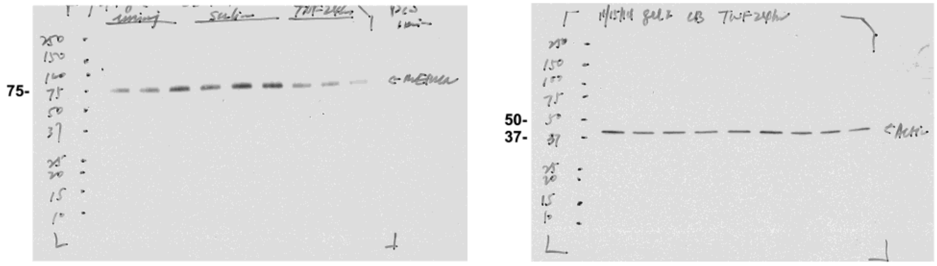

mEMCN Actin

Supplementary Figure 6. Full scans of uncropped immunoblots.

Supp Fig. 1a

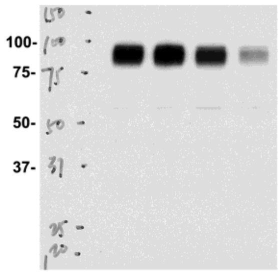

EMCN

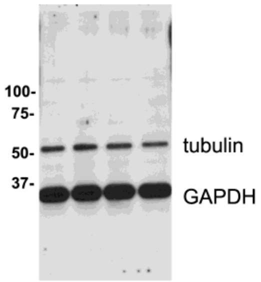

Tubulin/GAPDH

Supp Fig. 1b

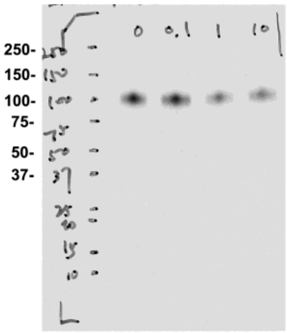

EMCN

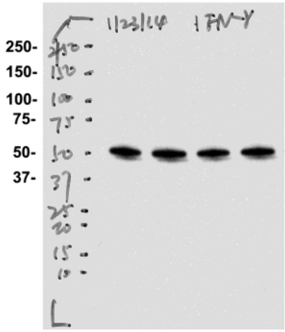

Tubulin

Supp Fig. 2a

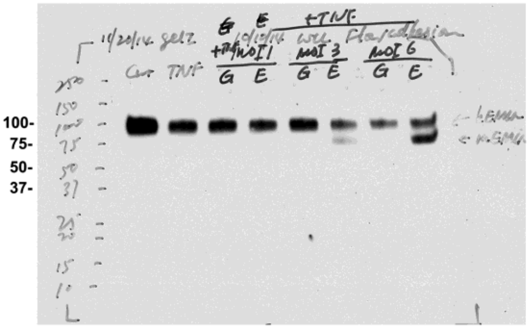

hEMCN/mEMCN

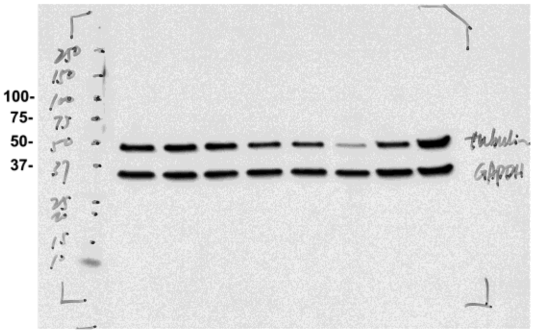

Tubulin/GAPDH

Supplementary Figure 6 – continued

Supp Fig. 3

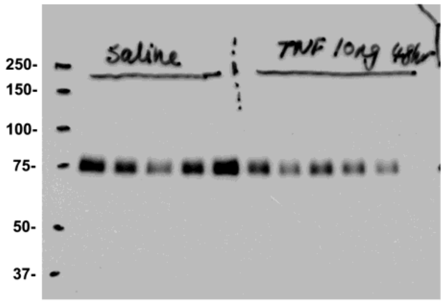

mEMCN

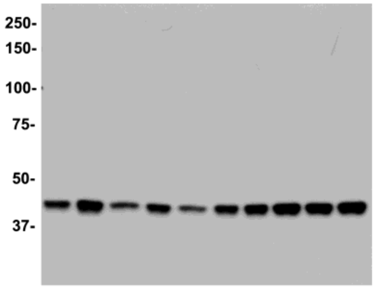

Actin

Supp Fig. 5a

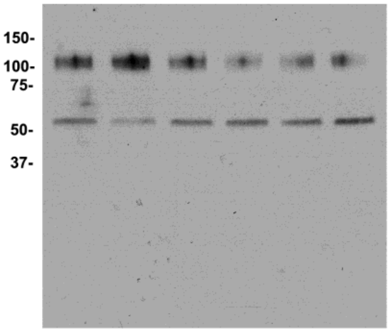

EMCN CS

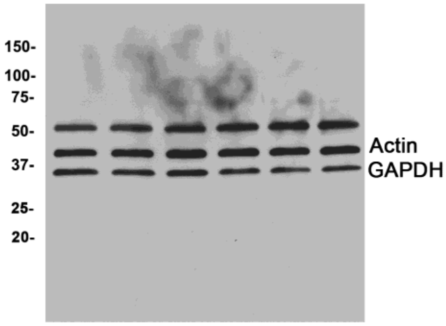

Actin/GAPDH

Supp Fig. 5b

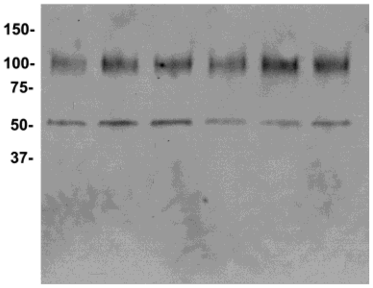

EMCN CS

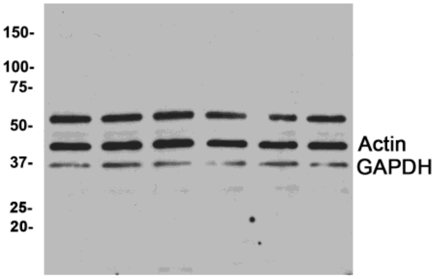

Actin/GAPDH

Supplementary Figure 6 – continued
